# Case report: COVID-19-associated refractory thrombotic thrombocytopenic purpura complicated with Guillain-Barré syndrome

**DOI:** 10.3389/fneur.2023.1199889

**Published:** 2023-05-24

**Authors:** Rui Zhou, Shenjian Chen, Qinghua Luo, Xinyue Zhang, Fang Li, Wei Huang, Zhiyong Sheng

**Affiliations:** ^1^Department of Neurological Intensive Care Unit, The Second Affiliated Hospital of Nanchang University, Nanchang, Jiangxi, China; ^2^Department of Neurology, The Second Affiliated Hospital of Nanchang University, Nanchang, Jiangxi, China; ^3^The Second Affiliated Hospital of Nanchang University, Nanchang, Jiangxi, China

**Keywords:** COVID-19, Guillain-Barré syndrome, thrombotic thrombocytopenic purpura, ADAMTS-13, rituximab

## Abstract

Thrombotic thrombocytopenic purpura (TTP), a rare and lethal thrombotic microangiopathy, is an autoimmune disease that can be triggered by viral infections such as COVID-19. This condition is characterized by hemolytic microangiopathy, thrombocytopenia, and neurologic alterations, possibly accompanied by fever and renal damage. Moreover, more than 220 patients with Guillain-Barré syndrome (GBS) have been reported in association with the COVID-19 infection. In this report, we present a case of a patient who developed refractory TTP complicated by GBS following a SARS-CoV-2 infection. We aimed to highlight the importance of accurately diagnosing neurological complications associated with a COVID-19 infection and to demonstrate our strategies for treating a patient with COVID-19 infection-related refractory TTP complicated by GBS.

## Introduction

It has been reported that SARS-CoV-2 infection may be complicated by a variety of immunological phenomena, including thrombotic thrombocytopenic purpura (TTP), Guillain-Barre syndrome (GBS), and autoimmune hemolytic anemia (1–4). Thrombotic thrombocytopenic purpura (TTP) is a thrombotic microangiopathy with high mortality. The formation of autoantibodies in the serum is the cornerstone, leading to a severe deficiency of ADAMTS-13 (a disintegrin and metalloproteinase with a thrombospondin type 1 motif, member 13), a Von Willebrand factor (vWF)-cleaved serine protease (5). ADAMTS-13 deficiency leads to the formation of ultra-large vWF with platelet aggregation, which can obstruct microvessels and result in predominant clinical manifestations, including microangiopathic hemolytic anemia (MAHA), thrombocytopenia, neurologic alterations, and possibly fever and renal damage. TTP is considered an emergency condition with high mortality, and early diagnosis and timely treatment are crucial for increasing the chances of survival. The PLASMIC score enables clinical diagnosis of TTP and guides early treatment based on medical history and laboratory findings (Table 1). A high PLASMIC score (6–7) indicates severe ADAMTS13 deficiency (≤10% activity) (6).

**Table 1:** The PLASMIC score.

**Parameter**	**Results of this patient**	**Score**
**Platelet count**		
<30 × 10^9^/L	7 × 10^9^/L	1
Hemolysis	6.11 mg/dL	1
Either: reticulocyte count > 2.5%		
Haptoglobin undetectable		
Indirect bilirubin > 2 mg/dL		
Presence of active cancer	No	1
History of organ or stem-cell transplant?	No	1
MCV <90 fL (9.0 × 10^−14^/L)	86.3 fL	1
INR <1.5	1.07	1
Creatinine <2.0 mg/dL	0.88 mg/dL	1

Guillain-Barré syndrome (GBS) is an acute autoimmune disease that affects the peripheral nerves and usually follows a viral or bacterial infection. Since the onset of the COVID-19 epidemic in December 2019, various studies have suggested that GBS may occur as a postinfectious pattern following a SARS-CoV-2 infection, be a part of the “long COVID-19 syndrome”, or be a form of para-infectious paralysis associated with the viral infection (7). GBS is characterized by rapidly progressive, distally onset symmetrical limb weakness, hyporeflexia, and sensory dysfunction, with or without respiratory or cranial nerve-innervated muscle involvement (8). Approximately 50% of patients' clinical symptoms peak within 2 weeks, and more than 90% develop to peak within 4 weeks.

There are case reports of GBS or TTP associated with the COVID-19 infection. In this study, we report on a case of sequential TTP and GBS following a COVID-19 infection for the first time. We successfully treated this 56-year-old male patient with 20 sessions of therapeutic plasma exchange and rituximab therapy.

## Case presentation

**Figure 1:**
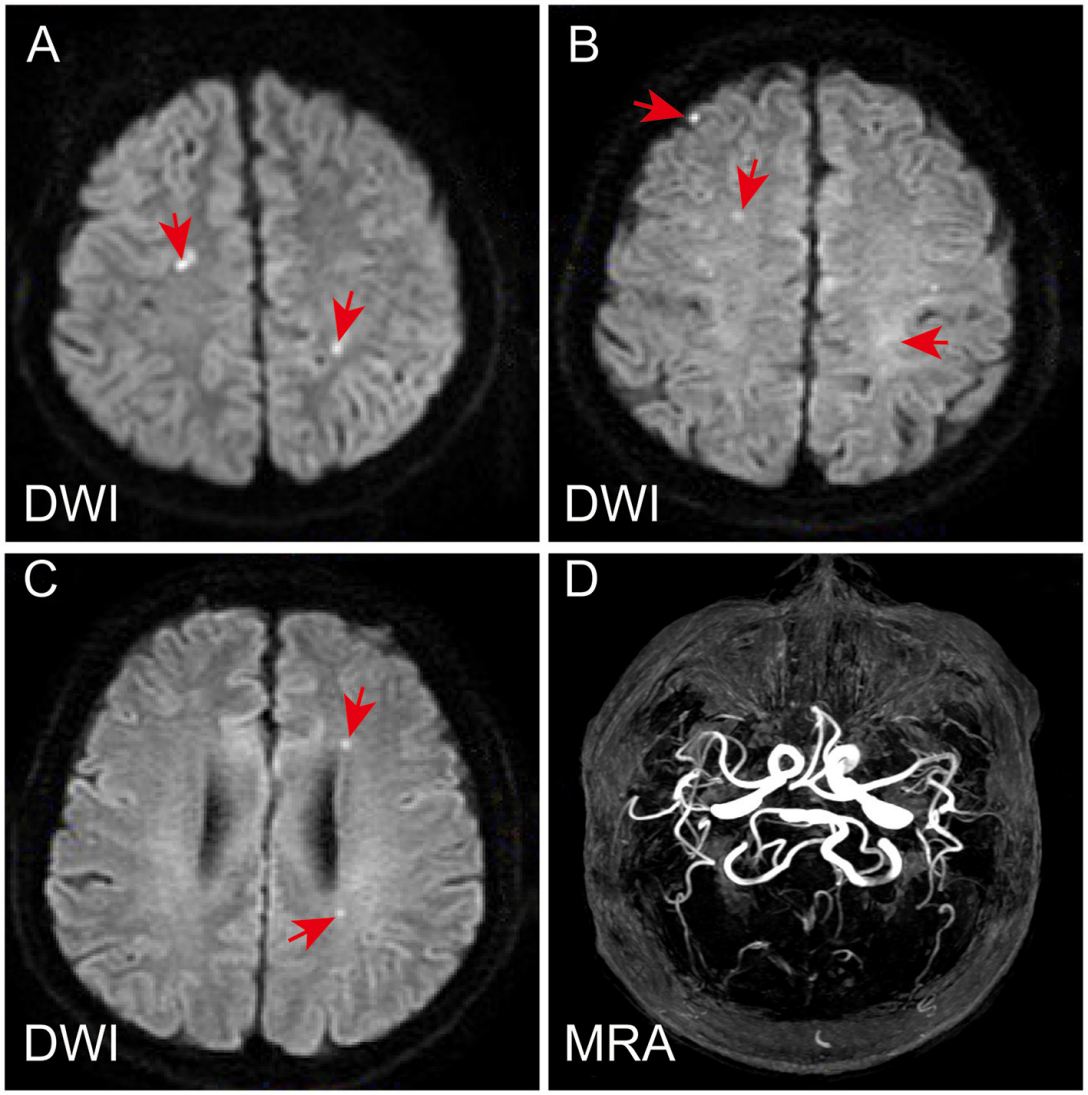
Magnetic resonance imaging and magnetic resonance angiography of the patient's brain. Diffusion-weighted (DWI) sequence revealed multiple acute or subacute lacunar infarcts in bilateral frontoparietal watershed areas **(A–C)**. Magnetic resonance angiography showed normal **(D)**.

**Figure 2:**
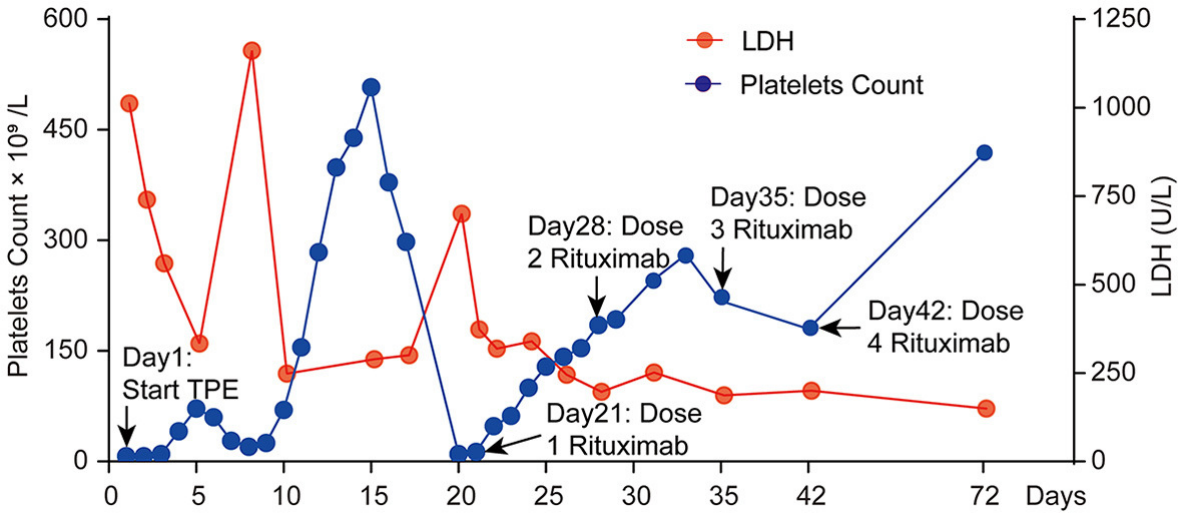
Clinical course of platelet count and LDH change in this patient during the first 72 days.

A 56-year-old male patient, who had a history of penicillin allergy and tested positive for COVID-19 2 weeks prior, presented to our hospital with acute episodes of headache for 2 days and a 13-h episode of gibberish. He had no significant past or family history of genetic or autoimmune diseases. Upon admission, his oropharyngeal swab for COVID-19 was detected as positive using the reverse transcription polymerase chain reaction (RT-PCR) method. Cranial MRI with magnetic resonance angiography revealed multiple acute or subacute lacunar infarcts in the bilateral frontoparietal watershed areas (refer to Figure 1). Chest CT scanning on admission revealed diffuse ground-glass-like opacity in the lower lobe of both lungs (Supplementary Figure S1). During the physical examination, the patient was found to be unconscious and agitated, with dull pupillary light reflexes on both sides and respiratory failure. Laboratory investigations showed mild anemia (hemoglobin 114 g/L), severe thrombocytopenia 7 × 10^9/^L (normal value: 100–300 × 10^9/^L) (Figure 2), high levels of total bilirubin 6.95 mg/dL (normal value: 0–1.35 mg/dL) and indirect bilirubin 6.11 mg/dL (normal value: 0–1.11 mg/dL) with normal serum alanine transaminase (ALT) and aspartate aminotransferase (AST), lactate dehydrogenase (LDH) 1,011.88 U/L (Figure 2), normal blood creatinine and urea nitrogen, high levels of D-dimer (1,130 ng/mL), and a clear increase in ferritin > 1,650 ng/mL. Serologic cytokine testing revealed a sharp rise in IL-6 at 109.09 pg/mL (normal value: 0–5.4 pg/mL) and in IL-8 at 240.97 pg/mL (normal value: 0–20.6 pg/mL). Arterial blood gas indicated acute hypoxic respiratory failure with a normal lactate level. Blood and sputum cultures were negative, and no other respiratory viruses were detected. All of these results strongly suggest a diagnosis of TTP. To rule out other causes of thrombocytopenic anemia, antinuclear antibodies (ANA) and antineutrophil plasma antibodies (ANCA) were also tested, both of which were negative. In conjunction with his medical history and laboratory results, the PLASMIC score for the patient was 7-point (Table 1), indicating a more than 80% probability of ADAMTS13 deficiency (9). Therefore, daily therapeutic plasma exchange (TPE) (1.5 volume exchange with cryo-poor plasma (CPP) as a replacement fluid) was started immediately, along with intravenous dexamethasone (15 mg) therapy. To confirm the diagnosis of TTP, ADAMTS13 levels were evaluated before TPE therapy, and the diagnosis of TTP was confirmed by a dramatic decrease in ADAMTS13 activity (0%) and strong positive anti-ADAMTS13 antibodies.

**Figure 3:**
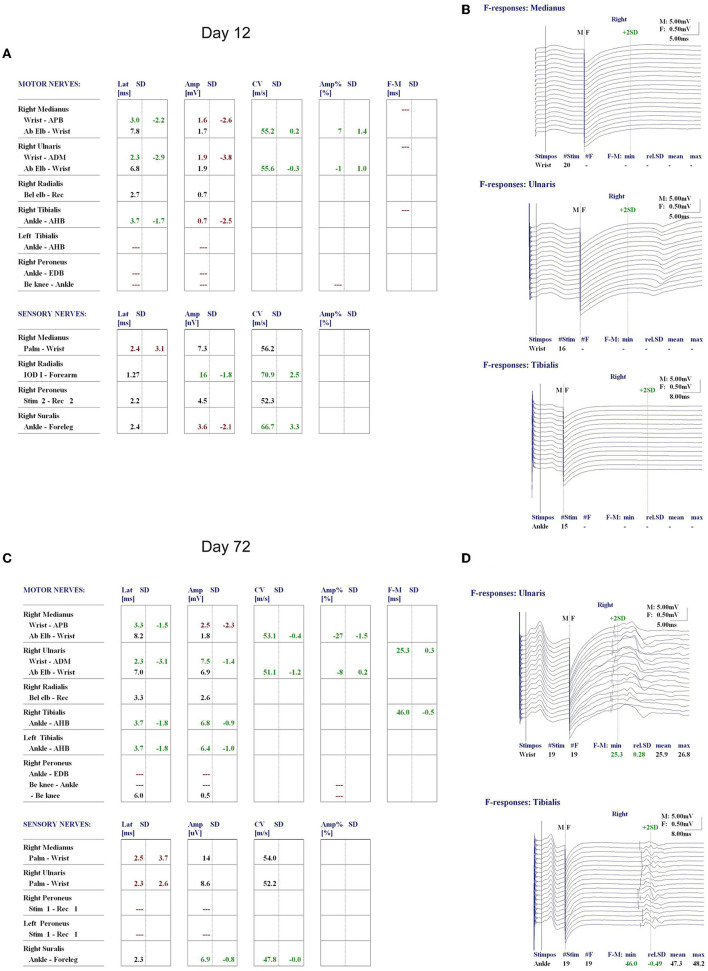
Nerve conduction study (NCS) of the patient on day 12 of admission showed a decrease in compound muscle action potential (CMAP) of all examined motor nerves **(A)**, and the F wave was absent in the median and tibial nerves **(B)**. Re-examination of NCS on day 72 revealed a significant improvement in the CMAP decrease in all motor and sensory nerves compared to that on day 12 of admission **(C)**, and normal F waves in the ulnar and tibial nerves were observed **(D)**.

On the 12th day of consecutive TPE and dexamethasone treatments, the patient's platelet count increased to 299 × 10^9^/L, and his LDH level decreased to 248.17 U/L (Figure 2). However, he remained unconscious and developed respiratory muscle weakness and ascending limb weakness with global areflexia. A follow-up cranial MRI showed no new abnormalities compared to those observed at admission. Given the patient's weakness in respiratory muscles and limb weakness with global areflexia, concomitant GBS was suspected. Nerve conduction studies confirmed the acute inflammatory demyelinating polyradiculopathy (AIDP) variant of GBS, which showed a decrease in compound muscle action potential (CMAP) of all examined motor nerves, with the F wave absent in the median and tibial nerves, while other testing revealed normal results (Figure 3). The nerve conduction studies demonstrated extensive peripheral nerve damage in the patient, with damage to motor axons. Lumbar puncture was also performed, and cerebrospinal fluid (CSF) analysis showed a protein level of 594.18 mg/L (normal values: 250–470 mg/L), while the white blood cell count was 5 × 10^6^/L (normal values: 0–8 × 10^6^/L), IL-6 was 78.56 pg/mL (normal value: 0–5.4 pg/mL), and IL-1β was 110.06 pg/mL (normal value: 0–12.4 pg/mL). The analysis of serum and CSF for antiganglioside antibodies showed negative results for anti-GQ1b, anti-GM1, anti-GM2, anti-GM3, anti-GD1a, anti-GD1b, and anti-GT1b. Additionally, serological tests for CMV, EBV, HIV, TP, HBV, and HCV antibodies were all negative.

On day 20 (day 7 after TPE discontinuation) of admission, platelet count declined sharply again to severe thrombocytopenia (10 × 10^9^/L), and LDH level also rose to 700.1 U/L. Refractory TTP was confirmed as an initial combination therapy failure, and symptoms were exacerbated. The patient was then started on four doses of rituximab injections (375 mg/m^2^ per week) while TEP therapy was restarted. After LDH levels were normal and platelet counts remained over 100 × 10^9^/L for two consecutive days, the frequency of plasma exchange was decreased to twice a week until terminated (total of eight TPE sessions), and the patient was fully conscious, no longer needed a ventilator, and could breathe normally.

Upon discharge on the 35th day of admission, the patient showed dramatic improvement in his ecchymosis, dyspnea, and limb weakness, but mild impairment in his proximal limbs remained with a power of 5-/5. Laboratory investigations showed that his hemoglobin was 119 g/L, platelet count was 218 × 10^9^/L, and his ALT, AST, blood creatinine, urea nitrogen, LDH, and bilirubin levels were all within normal ranges. A re-examination of the nerve conduction study on day 72 showed a significant improvement in CMAP decrease in all motor and sensory nerves compared to the previous one, with normal F waves observed in the ulnar and tibial nerves (Figure 3).

## Discussion and conclusions

TTP is a life-threatening thrombotic microangiopathy syndrome (TMA) that results from increased platelet adhesion and aggregation. Acquired TTP occurs due to the deficiency of ADAMTS13 caused by ADAMTS13 autoantibodies. Multiple studies have revealed that COVID-19 infection plays a role in triggering TTP. The pathogenesis of COVID-19-associated TTP might be related to cytokine storms and immune complexes that induce endothelial injury and produce ADAMTS13 inhibitors, which have been observed in autopsies of COVID-19 non-survivors (10). Platelets and endothelium express ACE2, a receptor for SARS-CoV-2 and its spike protein, and their binding results in enhanced thrombosis (3).

Moreover, the hallmarks of TTP, decreased ADAMTS13 activity, and highly elevated VWF have been reported as biomarkers that reflect intensive inflammation and endothelial damage (11). In some COVID-19 patients, SARS-CoV-2 RNA may provoke the production of IgG-type autoantibodies, which may interact with ADAMTS13 and lead to dysregulation of the VWF-ADAMTS13 axis, resulting in the accumulation of ultra-large VWF multimers, spontaneous aggregation of platelets, uncontrolled thrombosis in the microcirculation, and eventually, the patient developing TTP (12, 13). In this case, we found a strong positive result for the ADAMTS13 inhibitor in the context of COVID-19 infection.

In this case, the patient developed GBS as a complication of the COVID-19 infection and acquired TTP. Although testing for antiganglioside antibodies and other serologic tests for CMV, EBV, HIV, TP, HBV, and HCV were negative, the clinical presentation of respiratory and limb weakness with global areflexia could not be explained by the site of occlusion seen on cranial MRI. In light of previous reports of GBS as a common neuromuscular complication of COVID-19 infection, the patient was suspected of having a combination of GBS, which often occurs following respiratory or gastrointestinal viral infections. This conjecture was supported by nerve conduction studies showing extensive peripheral nerve damage (Figure 3). It is possible that the development of TTP and GBS, in this case, was due to a cross-reaction of antibodies produced against the SARS-CoV-2 virus, possibly resulting from molecular mimicry (14). It has been reported that other antiganglioside antibodies may also be present in GBS, such as anti-GD3, GT1a, GalNAc-GD1a, and GA1 (15). Therefore, it may be recommended to test for these antibodies when suspecting GBS, especially in cases of typical antiganglioside antibody negativity. Moreover, researchers have proposed an alternative explanation for the COVID-19 cases associated with TTP and GBS, suggesting that non-immunologic mechanisms, such as cytokine storms or microvascular disorders due to vascular endothelial injury, could be responsible (16). This patient presented with remarkably high inflammatory markers IL-6 and IL-8 in the serum, and increased IL-6 and IL-1β were found in the CSF, suggesting that non-immunologic mechanisms should be considered as a potential etiology of GBS in this patient.

TTP is a hematologic emergency with a 90% mortality rate if left untreated. In this case, the PLASMIC score was used for the initial diagnosis of TTP and the evaluation of the ADAMTS13 deficiency. The PLASMIC score of this patient was 7-point, indicating severe ADAMTS13 deficiency (activity ≤10%) and necessitating urgent and optimal treatment. TPE can predict prognosis, and failure of the platelet count to rise above 150 × 10^9^/L after seven consecutive TPE sessions indicates refractory TTP. The platelet count was the primary parameter used to assess the treatment response. In this case, the platelet count increased slightly after the patient received TPE and corticosteroid therapy but then dropped again and eventually stabilized above 150 × 10^9^/L after 12 consecutive TPE sessions. Unfortunately, the platelet count declined to 10 × 10^9/^L just 1 week after TPE termination, indicating unresponsiveness to TTP treatment (17).

Rituximab is an anti-CD20 monoclonal antibody that targets B lymphocytes and is the most effective treatment for refractory or relapsed TTP. Clinical studies report that treatment with rituximab alone resulted in clinical remission in 87% to 100% of acutely refractory and relapsing TTP patients, with platelet recovery within a median of 11 to 14 days after the first dose (18, 19). The American Society of Hematology recommends that in COVID-19-associated TTP, rituximab administration should be delayed until after the acute infection phase of COVID-19, as rituximab use may be associated with viral reactivation and increased compromise of the primary viral infection (13). This patient was administered rituximab after confirming that his oropharyngeal swab COVID-19 RT-PCR was negative. The platelet count recovered to 150 × 10^9^/L within 7 days after the first dose of rituximab, with an improvement in clinical symptoms. Current data suggest that rituximab may increase ADAMTS13 activity, reduce anti-ADAMTS13 autoantibodies, and likely be beneficial in preventing relapse (18).

Watershed infarcts are a type of stroke that occurs in the border zones between territories where the blood vessels from two different arteries converge. They are directly caused by reduced cerebral blood flow, which is mainly associated with atherosclerosis, hypotension, and embolism (20). Our patient's watershed infarction was observed on MRI, which may be associated with TTP-induced thrombosis. Furthermore, COVID-19 infection significantly increases the risk of stroke by causing inflammation in the blood vessels and a hypercoagulable state, both of which can increase the risk of clot formation (21). In this case, the patient's IL-6 inflammatory factors and ferritin levels were substantially increased, and a COVID-19-related cytokine storm may have also been present. Therefore, the watershed infarcts observed in this patient may be closely related to their COVID-19 infection.

The successful treatment of our patient depends on a rapid diagnosis with PLASMIC risk stratification and timely initiation of TPE, along with corticosteroid and rituximab therapy. Platelet counts improved significantly on day 7 with rituximab in our patient, indicating that prompt commencement of rituximab treatment in refractory TTP relapses with poor response to TPE may improve the likelihood of survival. Moreover, after reviewing the literature, we realized that the relapse of TTP in this patient might be partly due to the abrupt cessation of TPE treatment only 2 days after the platelet count reached 150 × 109/L and the lack of TPE maintenance therapy. Although the debate regarding whether TPE sessions should be terminated immediately or tapered following TTP remission continues, this case indicates that a gradual reduction of TPE may be a more prudent approach to prevent sudden exacerbations.

## Data availability statement

The datasets presented in this article are not readily available because of ethical and privacy restrictions. Requests to access the datasets should be directed to the corresponding authors.

## Ethics statement

The studies involving human participants were reviewed and approved by Institutional Review Board and Ethics Committee of the Second Affiliated Hospital of Nanchang University. The patients/participants provided their written informed consent to participate in this study. Written informed consent was obtained from the individual(s) for the publication of any potentially identifiable images or data included in this article.

## Author contributions

ZS and WH examined the patient and used the treatment strategy. RZ, SC, XZ, and FL acquired and analyzed all the clinical data. QL and ZS reviewed the literature and drafted the manuscript. WH supervised the study. All authors contributed to the article and approved the submitted version.

## Supplementary material

Supplementary Figure S1 Chest CT scanning findings in the patient. A diffuse ground-glass-like lesion in the lower lobe of both lungs.
