# Supplementary figures and images for: Case report: COVID-19-associated refractory thrombotic thrombocytopenic purpura complicated with Guillain-Barré syndrome
Source: Front Neurol. 2023 May 24;14:1199889. doi: 10.3389/fneur.2023.1199889 (PMC10244661; doi:10.3389/fneur.2023.1199889)

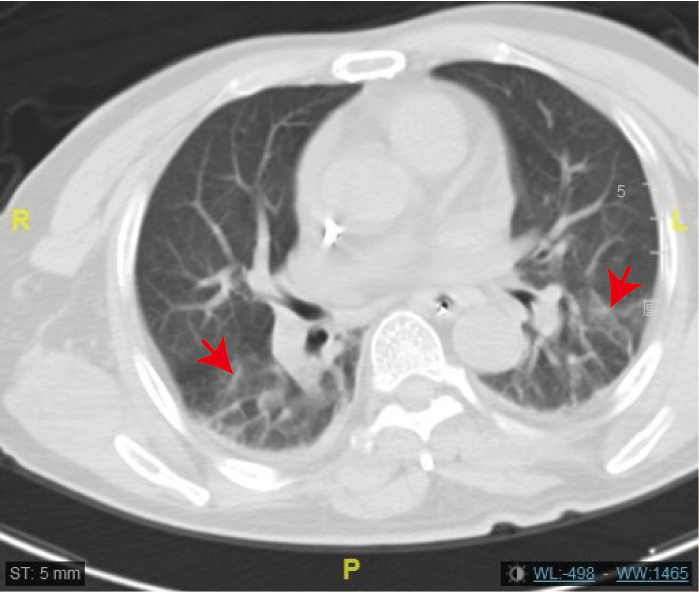

Supplement: Supplementary Figure S1 — Chest CT scanning findings in the patient. A diffuse ground-glass-like lesion in the lower lobe of both lungs. [file Image_1.TIF]
